# Cardiovascular Safety Evaluation of Febuxostat and Allopurinol: Findings from the FDA Adverse Event Reporting System

**DOI:** 10.3390/jcm12186089

**Published:** 2023-09-20

**Authors:** Yang Bai, Bin Wu, Liangwen Gou, Zhenwei Fang, Ting Xu, Tiejun Zhang, Yuwen Li

**Affiliations:** 1 Department of Pharmacy, West China Hospital, Sichuan University, Chengdu 610041, China; baiyang@wchscu.cn (Y.B.); binw83@hotmail.com (B.W.); tingx2009@163.com (T.X.); 2 West China School of Pharmacy, Sichuan University, Chengdu 610041, China; 3 Department of Neurosurgery, West China Hospital, Sichuan University, Chengdu 610041, China

**Keywords:** febuxostat, allopurinol, FAERS, cardiovascular toxicity, adverse event

## Abstract

Background: Febuxostat and allopurinol are the most commonly used uric acid-lowering medications, and their safety is of great concern, especially the cardiovascular adverse reactions associated with febuxostat. We propose to study the cardiovascular toxicity of febuxostat and allopurinol using the FDA Adverse Event Reporting System (FAERS) database. Methods: A total of 64 quarters of FAERS data were downloaded from 2004 to 2019. Febuxostat- and allopurinol-related cardiovascular adverse events were extracted after data cleaning. Signal detection was conducted by reporting odds ratio (ROR) and proportional reporting ratio (PRR). Results: There were 2939 and 25,219 reports of febuxostat- and allopurinol-related cardiovascular adverse events (CVAEs), respectively. The most frequent CVAEs with febuxostat and allopurinol were edema peripheral (14.38%) and peripheral swelling (8.76%), respectively. In elderly gout patients, febuxostat is associated with an increased risk of heart failure, ischemic heart disease, hypertension, and cardiomyopathy. Febuxostat in combination with acetic acid derivatives nonsteroidal anti-inflammatory drug (NSAIDS) also increases the risk of cardiovascular adverse events. Conclusions: Compared with allopurinol, febuxostat may increase cardiovascular toxicity in patients with gout.

## 1. Introduction

Gout is a common chronic disease that typically manifests itself as redness, swelling, and pain in the joints [1]. Lowering uric acid levels in the body is an important treatment for gout [2]. Allopurinol has been used in clinical practice for more than 50 years and it has been a commonly prescribed drug for the treatment of gout [3]. Allopurinol is a hypoxanthine analog that inhibits the conversion of hypoxanthine and xanthine to uric acid by interfering with the enzyme xanthine oxidase [4], thereby reducing uric acid concentrations [5]. However, the use of allopurinol may cause adverse reactions, including toxic epidermal necrolysis syndrome and Steven John’s syndrome, which are usually thought to be related to time, drug concentration, and genetics [6]. An association between HLA-B*5801 and an increased risk of allopurinol-associated serious allergic reactions was identified in a Chinese Han population [7]. The Clinical Pharmacogenomics Implementation Consortium currently recommends that HLA-B*5801-positive individuals avoid allopurinol [8]. In particular, the starting dose of allopurinol needs to be adjusted in patients with renal impairment [9]. These overshadow the use of allopurinol.

Febuxostat is a late-marketed, selective xanthine oxidase inhibitor, developed after allopurinol [10]. Several studies have shown that no dose adjustment is required in mild to moderate hepatic or renal impairment, or in elderly patients [11]. Currently, the U.S. gout guidelines recommend allopurinol as a first-line agent for all patients, including those with moderate-to-severe CKD [2], whereas in China, both febuxostat and allopurinol have been recommended as first-line agents for the treatment of gout [12].

However, the FDA placed a black box warning on febuxostat in 2019, because of its ability to cause a rise in heart-related and all-cause deaths [13]. A risk correlation between febuxostat and atrial fibrillation was also confirmed in a cohort study based on US Medicare data [14]. Conversely, there are also a large number of studies that have concluded that the safety and efficacy of febuxostat and allopurinol are comparable [15] or even better [16], especially in Asian populations [17].

Two of the largest studies to date—the CARES trial and the FAST trial—also disagreed on the cardiovascular safety of febuxostat. The CARES trial, published in the New England Journal of Medicine, concluded that febuxostat was noninferior to allopurinol in terms of the incidence of adverse cardiovascular events. However, all-cause mortality and cardiovascular mortality were higher with febuxostat than with allopurinol. These results were based on gout patients with comorbid major cardiovascular disease [18]. In contrast, the FAST trial demonstrated that the long-term administration of febuxostat did not increase the risk of death or serious adverse events compared with allopurinol [19]. Thus, the evidence for an association between febuxostat and adverse cardiovascular events remains equivocal.

The FAERS database is one of the most widely used adverse drug event reporting systems, and these data have been used in many high-quality studies to identify and evaluate adverse drug reactions after the introduction of drugs to the market [20,21].

In some meta-analyses, the cardiovascular safety conclusions for febuxostat have varied. For example, although febuxostat was not inferior to allopurinol in terms of cardiovascular risk, it did not reduce the incidence of gout [22,23]. Some views suggest that this may be related to the type of studies that the authors included in their analysis [24]. There are also analyses based on insurance databases that suggest febuxostat reduces all-cause mortality rates [25]. However, this study was based on data from only one country.

Currently, there is no conclusive statement on whether febuxostat shows cardiovascular toxicity, and we do not have a clear understanding of its biological mechanisms and clinical effects. Therefore, the aim of our study is to investigate the cardiovascular toxicity of febuxostat and another uric acid-lowering drug, allopurinol, by exploring the FAERS database to inform safe clinical use protected from these real-world adverse events.

## 2. Materials and Methods

### 2.1. Data Source, Extraction and Processing

The FDA Adverse Event Reporting System (FAERS) is a spontaneous reporting system that contains all adverse event information and medication error information collected by the FDA, and is free to the public. We downloaded all FARES database information from the first quarter of 2004 through the fourth quarter of 2019. We chose the tables of patient demographic and administrative information (DEMO), MedDRA terms for adverse event (REAC) and drug/biologic information (DRUG) for the subsequent analysis.

Although most of the reports submitted are from the United States, they can be submitted by any country. So there may be inconsistencies in the names of some drugs and adverse events (AEs). AEs and medication errors were coded using terms in the Medical Dictionary for Regulatory Activities (MedDRA) terminology [26]. In this study, MedEx_UIMA_1.3.7, developed by Vanderbilt University, was used for the standardization of drug names [26]. MedDRA version 21.1 was used for Preferred Terms (PTs) coding and language localization. To avoid duplicate reports, we identified and eliminated duplicate reports from the DEMO table based on FAERS documentation. In addition, in this study, which included an analysis of a restricted indication (gout), we selected only reports submitted by medical professionals, including physicians, pharmacists, and other health professionals, to ensure the reliability of the results.

### 2.2. Visualization of Data

The sex and age ratio of reported cases and number of reported cases per year were processed for plotting using excel sheet. The occupations of the reporters and the countries of reporting were plotted by https://www.bioinformatics.com.cn (last accessed on 10 July 2023), an online platform for data analysis and visualization. The occupations of the reporters were visualized using a proportional area chart map. The world heat map was used for data visualization for the top 7 countries in terms of number of reports.

The structural formula of febuxostat and allopurinol was drawn by KingDraw (Version 4.0.0)

### 2.3. Statistical Analysis

This retrospective pharmacovigilance study used disproportionality analysis, a well-established method that helps to detect signals from the FAERS database [27]. Disproportionality analysis is used to assess whether the observed number of drug–AE combinations is higher than the expected number [28]. The expected number is the incidence of AE for other drugs in the entire database. Exceeding a preset threshold signaled risk. In this study, all databases were analyzed using the reporting odds ratio (ROR) with the following statistical signaling criteria: lower 95% confidence interval ≥ 1, case number ≥ 3 [29], and proportional reporting ratio (PRR), where a signal was detected if case number ≥ 3, PRR ≥ 2 and χ2 ≥ 4 [30].

Demographic data and differences in adverse cardiovascular events between febuxostat and allopurinol, as well as interactions between febuxostat and NSAIDS, were investigated using chi-square tests and odds ratio tests. All tests were two-sided, and *p*-values ≤ 0.05 were considered significantly different [28]. All data categorizations and statistics were performed using Microsoft Excel version 2020.

## 3. Results

### 3.1. Study Population

**Figure 1:**
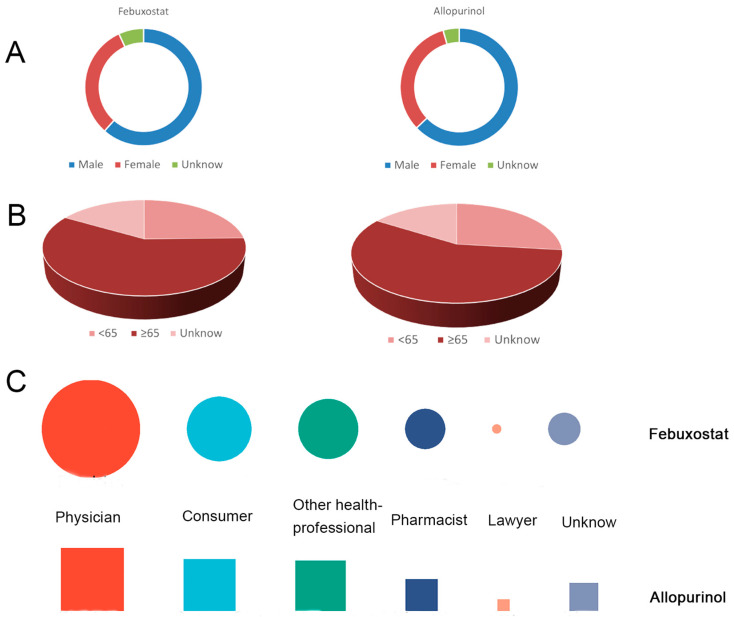
Basic information for reporting adverse cardiovascular events in FAERS. (**A**) Gender ratio of men and women in reported events. (**B**) Age distribution in reported events. (**C**) Report occupational information about the events. Visualized by a proportional area chart map. A larger area represents a larger number of reporters.

**Figure 2:**
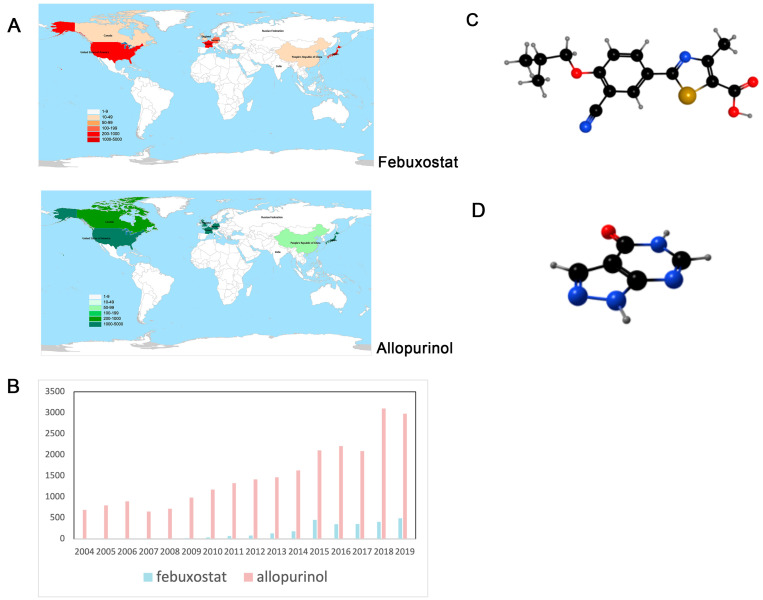
Regional overview and temporal variation of adverse cardiovascular events associated with febuxostat and allopurinol. (**A**) Country distribution of cardiovascular adverse events for both drugs, with only the top 7 countries identified. Darker colors represent a higher number of reports. (**B**) Changes in cardiovascular adverse events over time for two drugs, red for allopurinol and blue for febuxostat. (**C**) Structural formula of febuxostat. (**D**) Structural formula of allopurinol.

There were 2939 and 25,219 reports of adverse cardiovascular events related to febuxostat and allopurinol, respectively, in the FAERS database. Of these reports, valid demographic information related to febuxostat and allopurinol was reported in 2601 and 24,214 reports, respectively. Demographic information is shown in Table 1. There was a higher proportion of males than females among the cases of CVAEs (Figure 1A). More than 50% of the cases were older than 65 years (Figure 1B). CVAEs-related cases were mostly reported by physicians (febuxostat: 48.64%; allopurinol: 35.89%) and consumers (febuxostat: 20.65%; allopurinol: 24.19%). Allopurinol was reported most frequently in the United States, but febuxostat was reported most frequently in Japan (Figure 2A).

Overall, the use of allopurinol was reported in significantly higher numbers than febuxostat (Figure 2B). We noted a general upward trend in the number of cases of febuxostat over time (24 cases in 2009 and 491 cases in 2019), but a sharp increase in 2015. A similar trend was seen in the number of cases of allopurinol (691 cases in 2004 and 2976 cases in 2019). Although the number of febuxostat-associated CVAEs was much lower than that of allopurinol, the proportion of CVAEs for both drugs as a percentage of all their adverse events was about 25%.

### 3.2. Signaling of Cardiovascular Adverse Events with Febuxostat

We analyzed all 2939 reports related to febuxostat. A total of 14,696 adverse events were reported, including 2706 CVAEs (Supplementary Table S1). The CVAEs shown in Table 2 occurred with a frequency greater than 1%, which is indicative of common adverse reactions. Table 2 summarizes the positive results of the 22 disproportionality analyses. The top 5 CVAEs with the highest number of reported cases were peripheral edema, heart failure, atrial fibrillation, congestive heart failure, and edema. Heart failure showed the strongest signal (PRR, χ2, and ROR95% CI: 6.42, 1774.31, and 5.94). The next strongest signal was for chronic heart failure (PRR, χ2 and ROR95%CI: 23.21, 1135.24 and 17.80). In addition, an important CVAE to be emphasized is sudden death (1.40%), which showed a signal in both methods.

### 3.3. Signaling of Cardiovascular Adverse Events with Allopurinol

We analyzed a total of 25,219 reports related to allopurinol. These reports contained 110,800 adverse events, including 25,967 CVAEs (Supplementary Table S2). Table 3 summarizes the positive results of the 21 disproportionality analyses, all of which also had an incidence rate of >1%, which is generally considered to be a high incidence rate. The top five CVAEs with the highest number of reported cases were peripheral swelling, atrial fibrillation, heart failure, congestive cardiomyopathy, and syncope.

Compared with febuxostat (Table 2), allopurinol was associated with more syncope, altered mental status, electrocardiographic QT prolongation, and cardiomegaly, but a lower rate of sudden death.

### 3.4. Febuxostat Is Associated with an Increased Risk of Heart Failure, Ischemic Heart Disease, Hypertension, and Cardiomyopathy in Patients with Gout

To examine the cardiovascular safety of febuxostat and allopurinol in patients with gout, we selected 8687 febuxostat-associated AEs and 52,571 allopurinol-associated AEs, all of which occurred in patients with gout. Furthermore, to ensure the accuracy of the adverse event outcome determination and to reduce analytic error, we excluded inconclusive reports reported by consumers, lawyers, and uninformed persons. Ultimately, 4939 AEs associated with febuxostat and 27,310 AEs associated with allopurinol were included in the analysis.

We found that febuxostat had the most pronounced difference in causing ischemic heart disease and cardiac failure when compared with allopurinol, with the risk of ischemic heart disease being twice as high with febuxostat as with allopurinol. In addition, febuxostat also had a significantly higher risk of causing cardiomyopathy and hypertensive than allopurinol. There were no differences between febuxostat and allopurinol in terms of cardiac arrhythmias and torsade de pointes/QT prolongation (Table 4).

### 3.5. Febuxostat Is Associated with a Higher Risk of Cardiovascular Adverse Events in Elderly Patients with Gout

We also investigated whether the cardiovascular adverse events of febuxostat and allopurinol were associated with the age of patients with gout. We included 3185 reports of gout patients using febuxostat and 11,983 reports of gout patients using allopurinol. Again, inconclusive reports from consumers, lawyers, and unknowns were excluded. Ultimately, 1927 reports of febuxostat and 6494 reports of allopurinol were included in this analysis.

There were some differences in the demographic characteristics of those who experienced any CVAE after febuxostat administration and those who did not. Demographic information on the use of febuxostat is presented in Table 5. The mean age of those who had experienced any CVAE was 2 years older than the mean age of those who had not experienced a CVAE (68.20 versus 65.79, *p* = 0.007). The proportion of patients who had experienced a CVAE was higher in the older age group (age > 65 years). However, different sexes and different administered doses were not associated with having had a CAVE (Table 5).

In addition, after allopurinol administration, age, sex, and administered dose were not significantly associated with the occurrence of CVAE *(*Table 6).

### 3.6. Concomitant Use of Acetic Acid Derivatives NSAIDS Increased the Incidence of Cardiovascular Events

Patients with gout often require NSAIDs for pain relief. We also discussed whether the combination of drugs for gout with NSAIDs affects the occurrence of adverse cardiovascular events. There were a total of 166 reports of the coadministration of febuxostat with NSAIDs (Table 7), of which 91 (4.72%) were propionic acid derivative analogs, including: naproxen, ketoprofen, flurbiprofen, and ibuprofen; 46 were acetic acid derivative analogs (2.39%) including indomethacin and diclofenac. Selective COX-2 inhibitors comprised 29 copies (1.50%), including celecoxib, rofecoxib, etoricoxib, and romecoxib.

In reports of the coadministration of febuxostat with NSAIDs, the combination of acetate derivatives increased the reported rate of CVAE (3.75% vs. 2.03%, respectively; *p* = 0.045). However, there was no difference in the occurrence of CVAE with or without the combination of propionic acid derivatives or selective COX-2 inhibitors.

## 4. Discussion

In this study, we found that more than 50% of the patients who experienced adverse cardiovascular events after taking febuxostat and allopurinol were over 65 years of age. This suggests that we need to be concerned about the risk of cardiovascular disease in older patients taking these two drugs. But there are other reasons that should not be overlooked. Gout is more common in older patients [31], and older patients are at higher risk of CVD compared to younger people [32]. From the source of the reporters, the proportion of pharmacists is much smaller than that of physicians, suggesting that pharmacists should strengthen the monitoring of adverse reactions during the use of febuxostat and allopurinol. The United States has the highest number of cardiovascular events for allopurinol, which may be due to the fact that the FAERS system is the most developed and sophisticated in the United States [33]. Interestingly, Japan had the highest number of cardiovascular events with febuxostat. This may be because Japan is the country of origin of febuxostat [34]. In addition, China, a country with a large and aging population [35], reported fewer numbers. Increased pharmacovigilance is recommended for healthcare professionals in China.

Through data mining, we identified many new cardiovascular adverse events that far exceeded the adverse reactions documented in the inserts of both drugs, expanding our understanding of the cardiovascular risks of both drugs. Most cardiovascular adverse events were similar for both drugs. Peripheral edema, heart failure, and atrial fibrillation were the top three cardiovascular adverse events for febuxostat and allopurinol, which also showed strong positive signals (Table 2 and Table 3). Whereas Echocardiogram abnormal with febuxostat are not common adverse events (Supplementary Table S1), they show a strong positive signal. Changes in myocardial structure and function can often be detected by imaging before the onset of cardiac symptoms [36]. Therefore, if a patient develops echocardiographic abnormalities while taking febuxostat, we need to assess his or her risk in relation to febuxostat. More importantly, our analysis found that febuxostat was twice as likely to cause sudden death as allopurinol (Table 2).

Febuxostat and allopurinol are currently the first-line uric acid-lowering drugs, and we are more concerned about their safety in gout patients, so we further analyzed the cardiovascular adverse events in gout patients. The results found that febuxostat was more likely than allopurinol to cause adverse events such as heart failure, ischemic heart disease, hypertension and cardiomyopathy in gout patients. In the CARES study, the risk of death from any cause and the risk of cardiovascular death were higher in the febuxostat group than in the allopurinol group. Of these, sudden cardiac death was the most common cause of cardiovascular death [18]. These two well-known clinical studies reached different conclusions regarding the cardiovascular safety of febuxostat and allopurinol, which may be related to the different clinical endpoints of each study. In addition, the two studies recruited patients from different countries and regions [18,19].

In our study, the occurrence of heart failure was found to be significantly higher in patients treated with febuxostat than with allopurinol. Heart failure is one of the most important causes of cardiac death [37]. Our study’s findings were similar to the results of CARES. Special attention should be paid to the occurrence of cardiovascular adverse events when using febuxostat in gout patients.

By analyzing the basic information of gout patients who had used both drugs, we found that older adults taking febuxostat were more likely to experience adverse cardiovascular events. However, there was no difference for allopurinol. This suggests that we should pay special attention to elderly gout patients treated with febuxostat. However, we should not lose sight of the fact that age itself is an independent factor in cardiovascular disease [38]. Cardiovascular disease has traditionally been regarded as a “man’s problem” because the prevalence of cardiovascular disease is higher in men than in women [39]. However, in our findings, men taking two drugs for gout did not show a higher rate of adverse cardiovascular events. We analyzed the effects of different doses on the incidence of adverse cardiovascular events based on the maximum dose recommended in the drug insert. In general, larger doses of drugs are more likely to cause adverse events. However, in our results, there was no difference in adverse cardiovascular events between the high-dose and low-dose groups.

The US gout guidelines strongly recommend the use of anti-inflammatory drugs when starting uric acid-lowering therapy [2]. Therefore, combinations of drugs are very common in the treatment of gout. Among them, the combination of NSAIDs requires special attention [40]. In this study, we found that the combination of febuxostat and acetate derivatives was associated with an increase in adverse cardiovascular events compared with other NSAIDs. However, propionic acid derivatives (e.g., naproxen and ibuprofen) and selective COX-2 inhibitors did not increase the risk of cardiovascular events. NSAIDs have cardiovascular risks of their own, and one of the possible reasons for this is the observed shift in the prothrombotic–antithrombotic balance on endothelial surfaces towards thrombosis [41,42].Several retrospective analyses and meta-studies have concluded that both selective and non-selective NSAIDS may increase the incidence of cardiovascular adverse events, including myocardial infarction and myocardial ischemia [43,44], with diclofenac sodium (belonging to acetic acid derivatives) associated with more adverse events [45], which is similar to our findings. The U.S. gout guidelines do not recommend specific drugs for NSAIDs, but the results of this study suggest that it is safer to choose over-the-counter NSAIDs, such as ibuprofen, in patients receiving febuxostat for gout.

There are several limitations to our study. First, this study was based on a spontaneous database of adverse event reports, and information reported by laypersons may have been incorrect or missing. Second, reporters were influenced by external media reports and academic findings. When one aspect of a drug is perceived to be safer, that drug receives less attention. Therefore, the database is not representative of all drug use in the real world. In addition, only data from the database were statistically analyzed in this study, and the results were statistically significant. However, the causal relationship between medications and adverse events cannot be established, and further pharmacologic studies and higher-quality clinical studies are needed [46].

## 5. Conclusions

We are the first to mine the FAERS database to analyze cardiovascular-related adverse events related to febuxostat and allopurinol. Our study found that allopurinol had more cardiovascular adverse events than febuxostat, but febuxostat was associated with a higher risk of heart failure, ischemic heart disease, hypertension, and cardiomyopathy. Further, in older patients with gout, febuxostat can lead to a higher incidence of cardiovascular adverse events. We also found that patients had an increased risk of adverse cardiovascular events when febuxostat was combined with acetic acid derivatives NSAIDS. We believe that when using febuxostat, one should be concerned about its potential for cardiovascular adverse events.

## Figures and Tables

**Table 1:** Basic information for reporting febuxostat- and allopurinol-related adverse cardiovascular events in FAERS.

	Febuxostat	Allopurinol
Total number of reports	2939	25,219
Number of reports available	2601	24,214
Sex		
Male	1601 (61.55)	15,223 (62.87)
Female	821 (31.56)	7915 (32.69)
Unknow	179 (6.88)	1076 (4.44)
Age		
<65	641 (24.64)	6500 (26.84)
≥65	1528 (58.75)	13,836 (57.14)
Unknow	432 (16.61)	3878 (16.02)
Reporter		
Physician	1265 (48.64)	8691 (35.89)
Consumer	537 (20.65)	5857 (24.19)
Other health-professional	461 (17.72)	5536 (22.86)
Pharmacist	202 (7.77)	2163 (8.93)
Lawyer	8 (0.31)	266 (1.10)
Unknow	128 (4.92)	1701 (7.02)
Country		
Japan	1010 (38.83)	1879 (7.76)
United Sates	879 (33.79)	10,366 (42.81)
France	206 (7.92)	1354 (5.59)
Germany	152 (5.84)	1991 (8.22)
United Kingdom	46 (1.77)	1566 (6.47)
Canada	27 (1.04)	643 (2.66)
China	19 (0.73)	78 (0.32)
Other countries	208 (8.00)	4559 (18.83)
Unknow	54 (2.08)	1778 (7.34)

**Table 2:** Associations of cardiovascular adverse events with febuxostat.

PT	*N* (%)	PRR	χ2	ROR	ROR 95% CI
Total number of reports = 2706					
Edema peripheral	451 (14.38)	2.21	303.33	2.25	2.05
Cardiac failure	389 (9.57)	6.42	1774.31	6.57	5.94
Atrial fibrillation	221 (8.17)	3.04	302.45	3.07	2.69
Cardiac failure congestive	220 (8.13)	2.60	216.62	2.62	2.29
Edema	119 (7.10)	2.80	138.05	2.82	2.35
Blood creatine phosphokinase increased	99 (4.40)	3.65	189.55	3.66	3.01
Arrhythmia	95 (3.66)	2.39	76.79	2.40	1.96
Angina pectoris	67 (3.51)	2.60	65.99	2.61	2.05
Ascites	67 (2.47)	2.86	80.85	2.87	2.26
Cardiac failure acute	67 (2.47)	15.62	899.30	15.69	12.31
Acute myocardial infarction	61 (2.25)	2.21	40.53	2.22	1.72
Cardiac failure chronic	55 (2.03)	23.21	1135.24	23.29	17.80
Multiple organ dysfunction syndrome	50 (1.84)	3.85	105.01	3.86	2.92
Orthostatic hypotension	45 (1.66)	3.40	76.04	3.41	2.54
Acute coronary syndrome	42 (1.55)	5.48	152.90	5.49	4.05
Pulmonary congestion	41 (1.51)	3.97	90.71	3.98	2.93
Sudden death	38 (1.40)	3.74	76.07	3.75	2.73
Ventricular tachycardia	37 (1.37)	2.74	40.84	2.75	1.99
Right ventricular failure	35 (1.29)	5.39	124.30	5.40	3.87
Troponin increased	32 (1.18)	6.84	158.36	6.86	4.84
Stress cardiomyopathy	29 (1.07)	8.35	185.64	8.36	5.80
Ventricular extrasystoles	28 (1.03)	3.06	38.67	3.06	2.11

**Table 3:** Associations of cardiovascular adverse events with allopurinol.

PT	*N* (%)	PRR	χ2	ROR	ROR 95% CI
Total number of reports = 25,967					
Peripheral swelling	2275 (8.76)	2.71	2407.17	2.74	2.63
Atrial fibrillation	2066 (7.96)	3.86	4241.72	3.92	3.75
Cardiac failure	1942 (7.48)	4.36	4846.20	4.42	4.22
Congestive cardiomyopathy	1542 (5.94)	2.44	1291.34	2.46	2.34
Syncope	1416 (5.45)	2.33	1058.34	2.35	2.23
Bradycardia	1069 (4.12)	3.44	1794.50	3.46	3.26
Edema	882 (3.40)	2.80	995.04	2.81	2.63
Pulmonary oedema	787 (3.03)	2.97	1003.90	2.99	2.78
Arrhythmia	621 (2.39)	2.09	347.13	2.10	1.94
Acute myocardial infarction	596 (2.30)	2.92	731.27	2.93	2.70
Coronary artery disease	530 (2.04)	2.16	324.39	2.17	1.99
Blood creatine phosphokinase increased	514 (1.98)	2.54	468.89	2.55	2.33
Ascites	467 (1.80)	2.68	480.08	2.69	2.45
Angina pectoris	466 (1.80)	2.43	383.29	2.43	2.22
Mental status changes	434 (1.67)	2.43	358.16	2.44	2.22
Orthostatic hypotension	389 (1.50)	4.00	843.50	4.01	3.62
Electrocardiogram QT prolonged	387 (1.50)	2.05	203.68	2.05	1.85
Cardiomegaly	380 (1.46)	4.27	914.22	4.28	3.86
Ventricular tachycardia	353 (1.35)	3.55	624.60	3.56	3.20
Multiple organ dysfunction syndrome	320 (1.23)	3.33	505.32	3.34	2.98
Ejection fraction decreased	260 (1.00)	3.28	400.08	3.29	2.91

**Table 4:** Differences in adverse event categories between febuxostat and allopurinol.

CVAEs	Febuxostat	Allopurinol	*p*-Value	OR [95% CI]
Total number of adverse events	4943	27,310		
Total number of reports	1927	6494		
Cardiac failure	125 (2.53%)	461 (1.69%)	<0.001	1.511 (1.24–1.85)
Ischemic heart disease	111 (2.25%)	284 (1.04%)	<0.001	2.186 (1.75–2.73)
Cardiomyopathy	100 (2.02%)	410 (1.50%)	0.007	1.355 (1.09–1.69)
Cardiac arrhythmias	58 (1.17%)	334 (1.22%)	0.77	0.959 (0.72–1.27)
Hypertension	48 (0.97%)	189 (0.69%)	0.035	1.407 (1.02–1.93)
Torsade de pointes/QT prolongation	43 (0.87%)	277 (1.01%)	0.346	0.856 (0.62–1.18)

**Table 5:** Characteristics of patients administrated febuxostat.

	Total Number of Reports (*n* = 1927)	Any CVAE (*n* = 400)	Non-CVAE (*n* = 1527)	*p*-Value	OR [95% CI]
Age	1371 (71.15)	306 (76.50)	1065 (69.74)		
Mean (SD), range	66.33 (14.34), 6–98	68.20 (13.26), 26–98	65.79 (14.59), 6–97	0.007 ^a^	/
≥65	783 (40.63)	195 (48.75)	588 (38.51)	0.008 ^b^	1.426 (1.10–1.85)
<65	588 (30.51)	111 (27.75)	477 (31.24)		
Sex	1778 (92.27)	376 (94.00)	1402 (91.81)		
Male	1249 (64.82)	265 (66.25)	984 (64.44)	0.912 ^b^	1.014 (0.79–1.30)
Female	529 (27.45)	111 (27.75)	418 (27.37)		
Dose (mg)	1197 (62.12)	260 (65.00)	937 (61.36)		
≤80 (Recommended)	1170 (60.72)	254 (63.50)	916 (59.99)	0.949 ^b^	0.971 (0.39–2.43)
>80 (Overdose)	27 (1.40)	6 (1.50)	21 (1.38)		

^a^ Wilcoxon rank-sum test. ^b^ Chi-square test. CVAE: cardiovascular adverse event.

**Table 6:** Characteristics of patients administrated allopurinol.

	Total Number of Reports (*n* = 6494)	Any CVAE (*n* = 1453)	Non-CVAE (*n* = 5041)	*p*-Value	OR (95% CI)
Age	5631 (86.71)	1285 (88.44)	4346 (86.22)		
Mean (SD), range	68.47 (12.54), 13–105	68.16 (11.61), 22–96	68.57 (12.80), 13–105	0.065 ^a^	/
≥65	3705 (57.05)	819 (56.37)	2886 (57.25)	0.076 ^b^	0.889 (0.78–1.01)
<65	1926 (29.66)	466 (32.07)	1460 (28.96)		
Sex	6101 (93.94)	1393 (95.87)	4708 (93.40)		
Male	4285 (65.98)	999 (68.75)	3286 (65.19)	0.169 ^b^	1.097 (0.96–1.25)
Female	1816 (27.96)	394 (27.12)	1422 (28.21)		
Dose (mg)	3503 (53.94)	758 (52.17)	2745 (54.45)		
≤300 (Recommended)	3439 (52.96)	750 (51.62)	2689 (53.34)	0.073 ^b^	0.512 (0.24–1.08)
>300 (overdose)	64 (0.99)	8 (0.55)	56 (1.11)		

^a^ Wilcoxon rank-sum test. ^b^ Chi-square test. CVAE: cardiovascular adverse event.

**Table 7:** Combined use of febuxostat with nonsteroidal anti-inflammatory drugs.

	Total Number of Reports	Any CVAE	Non-CVAE	*p*-Value	OR [95% CI]
Concomitant drugs	166	41	125		
Propionic acid derivatives	91 (54.82)	21 (51.22)	70 (56.00)	0.576	1.153 (0.70–1.90)
Naproxen	48 (28.92)	14 (34.14)	34 (18.40)	0.146	1.593 (0.85–3.00)
Ibuprofen	36 (21.69)	7 (17.07)	29 (23.20)	0.845	0.920 (0.40–2.12)
Acetic acid Derivatives	46 (27.71)	15 (36.59)	31 (24.80)	0.045	1.880 (1.01–3.52)
Selective COX-2 inhibitor	29 (17.47)	5 (12.20)	24 (19.20)	0.638	0.793 (0.30–2.09)

CVAE: cardiovascular adverse event.

## Data Availability

The data presented in this study are available upon request from the corresponding author.

## Supplementary Materials

The following supporting information can be downloaded at: https://www.mdpi.com/article/10.3390/jcm12186089/s1, Table S1: Associations of cardiovascular adverse events with febuxostat. Table S2: Associations of cardiovascular adverse events with allopurinol.
